# Liquid Biopsy in Colorectal Cancer-Current Status and Potential Clinical Applications

**DOI:** 10.3390/mi9060300

**Published:** 2018-06-15

**Authors:** Gregor Norcic

**Affiliations:** Department of Abdominal Surgery, University Medical Centre Ljubljana, Zaloska Cesta 7, Ljubljana 1000, Slovenia; gregor.norcic@kclj.si; Tel.: +386-1522-7259

**Keywords:** colorectal cancer, circulating tumor cells (CTCs), circulating tumor DNA (ctDNA), liquid biopsy, lab-on-a-chip

## Abstract

Colorectal cancer is one of the most frequent solid malignancies worldwide. The treatment is either surgical or multimodal and depends on the stage of the disease at diagnosis. Accurate disease assessment is thus of great importance for choosing the most optimal treatment strategy. However, the standard means of disease assessment by radiological imaging or histopathological analysis of the removed tumor tissue lack the sensitivity in detecting the early systemic spread of the disease. To overcome this deficiency, the concept of liquid biopsy from the peripheral blood of patients has emerged as a new, very promising diagnostic tool. In this article, we provide an overview of the current status of clinical research on liquid biopsy in colorectal cancer. We also highlight the clinical situations in which the concept might be of the greatest benefit for the management of colorectal cancer patients in the future.

## 1. Introduction

Colorectal cancer (CRC) is one of the most frequent forms of solid cancer in the developed world. Although its incidence is stable or even slightly decreasing, CRC still presents a huge socioeconomic burden in these countries. Despite all of the efforts of modern medicine, the prognosis of patients with CRC mostly depends on the stage of the disease at diagnosis. In the United States, there were significant differences in the prognosis of patients diagnosed between 2001 and 2007 with localized tumors, regional tumor spread, and distant tumor spread, with the five-year survival of these patients being 90.1%, 69.2%, and 11.7%, respectively [1].

For decades, the management of CRC patients has relied on rather robust diagnostic methods such as histopathological analysis of tumor tissue. At the beginning of treatment, the histopathological analysis of a tumor tissue biopsy, obtained at colonoscopy, serves as a confirmation of the diagnosis. The analysis of the tumor tissue removed by surgery is of the greatest importance for the TNM staging of the disease, which itself is the basis for indicating adjuvant chemotherapy. A tissue biopsy of newly diagnosed lesions during the course of the follow-up of patients is often necessary in order to confirm the recurrence of the disease [1,2].

However, the clinical disease assessment and staging based on histopathological tumor tissue analysis, although prognostically significant for the population level, cannot provide clinically useful prognostic and predictive information for an individual patient’s level [2,3]. For this reason, clinical scientists are constantly looking for more reliable individual biomarkers. The presence of tumor traits in a patient’s blood stream has been identified as a potential individual biomarker with prognostic importance. At first, all of the attention was focused on the tumor cells found in the peripheral blood of patients, the so-called circulating tumor cells (CTCs). It has been shown in many studies that the detection of CTCs in the peripheral blood of patients has a negative impact on their survival [4,5,6]. Due to the near similarity with conventional tumor tissue biopsy, the term liquid biopsy has been introduced for CTC detection from blood probes [7]. Yet, it has turned out that not only the presence of CTCs but also the presence of circulating tumor DNA (ctDNA) is of clinical importance [8,9]. Furthermore, the biological role of exosomes in the pathogenesis of cancer is becoming evident and their presence in the peripheral blood of patients has also been proposed as an important biomarker [10,11,12,13]. Therefore, the term liquid biopsy has recently been broadened and is currently used for the detection of all tumor traits (CTCs, ctDNA, exosomes, microRNAs, and others) in the peripheral blood of patients [14,15,16,17,18].

The concept of liquid biopsy is slowly approaching clinical practice in the form of numerous clinical studies. In this article, we describe the potential clinical use of liquid biopsy on the case of CRC.

## 2. Potential Applications of Liquid Biopsy in Colorectal Cancer

### 2.1. Cancer Screening

Due to very good responses to treatment in the early stages of colorectal cancer, huge efforts have been invested in the early diagnosis of the disease in the developed countries. General- population screening programs are available in many European countries, mainly based on fecal occult blood testing, followed by colonoscopy in case of positive results [19]. Screening programs in the United States are more opportunistic, and scientific societies recommend interval colonoscopy (every 10 years) or annual fecal occult blood testing. The fecal occult blood tests employed are mainly immunohistochemical tests. For reasons of cost and efficacy, the DNA fecal tests, approved by the US Food and Drug Administration (FDA), have not been recommended as a first option [20].

Alternatively, somatic DNA mutations based blood tests have been suggested as a screening tool for colorectal cancer [21]. Indeed, many possible blood DNA screening markers have been identified, with only one test approved by the FDA for screening purposes in colorectal cancer so far [22,23]. Scientific recommendations, however, advise against such screening for the time being [20]. With recent advances in technology, a more effective liquid biopsy screening test might be developed in the near future [24].

### 2.2. Tumor Burden

CRC tumor burden at diagnosis is recognized as an important factor of disease assessment before the beginning of any treatment. In clinical terms, we are referring to a tumor stage. Tumor staging is thus an essential part of diagnostics and is performed immediately after the disease itself has been diagnosed. Clinicians currently rely mainly on radiologic imaging (computed tomography (CT) scan, magnetic resonance imaging (MRI), etc.) to assess the possible spread of the disease. These modalities are, however, quite inaccurate. Therefore, the final stage of the disease is often determined only after the beginning of treatment, that is, once the tumor has been surgically removed. The stage of the disease is eventually based on histopathological analysis of the resected specimen of the tumor and regional lymph nodes. The stage of the disease is reported in TNM categories according to AJCC/UCICC [25].

It is known from basic research that tumor cells spread from primary tumor into the bloodstream early in the course of tumor progression [26]. Although most of the CTCs do not survive very long in circulation, one could expect some CTCs in the peripheral blood of patients even in the early stages of solid tumors [27]. Therefore, the whole tumor burden might be estimated according to the number of CTCs in the peripheral blood of CRC patients of all tumor stages. Many researchers have already tried to find a correlation between either CTCs or ctDNA and the TNM stage of CRC. Some of them have found an association between tumor traits in the peripheral blood and tumor TNM stage [28,29,30,31]. The majority of researchers, however, have found no such correlation [32,33,34,35,36].

### 2.3. Residual Disease

Oncological (radical) tumor resection is currently regarded as the most effective treatment of CRC. It has been recognized for a long time that only the patients whose tumor tissue has been completely removed can expect to be cured. This complete removal has been termed R0 resection, meaning that there is no residual tumor tissue in a patient’s body after surgery, as opposed to R1 and R2 resections, where there is microscopic or macroscopic residual disease after surgery, respectively [37].

Based on common sense, one would expect that after a complete (R0) surgical removal there should be no signs of tumor in the patient’s blood. The detection of tumor traits in the peripheral blood might therefore be used to estimate the radicality of surgery. Some researchers have indeed tested the patient’s blood after an apparent curative surgery due to CRC for the presence of CTCs and ctDNA and have mostly reported a negative correlation between radical surgery and the postoperative presence of tumor traits in the peripheral blood [33,38,39]. Very few, however, have described the association of R category and tumor signs in the blood after surgery [34].

### 2.4. Prognostic Marker

The tumor burden (stage of the disease at diagnosis) and residual disease (R category) are generally accepted as the most important prognostic factors in CRC patients. The prognosis itself is expressed in the expected incidence of disease recurrence and the five-year survival of patients. Both parameters are usually reported for a specific tumor stage only (since it is significantly different for each of the four tumor stages) and for different R categories separately (since in cases of R1 and R2 resections, long term survival of patients cannot be expected). However, there are still no reliable factors to estimate the prognosis of individual patients within the specific prognostic group, although it is obvious from clinical practice that even the patients from the same prognostic group do not share the same prognosis.

The presence of CTCs/ctDNA in CRC patients has been studied as a prognostic factor in many clinical studies. Most of the authors report on the negative impact of tumor presence in the peripheral blood (positive tumor biopsy) before and/or after curative surgery on the incidence of disease recurrence and the patient’s survival [30,31,32,40,41,42]. Very few researchers have found no such correlation [43]. The negative impact of CTC presence in the peripheral blood has been observed even in the presence of a known metastatic disease [44]. The prognostic importance of CTCs, as well as ctDNA, has also been confirmed in the reviews of published literature [4,5,45]. Yet, the methodology, detection methods, cut-of values, etc. of all these clinical studies were much too different to enable us to draw any practical conclusions from their results on an individual patient’s level.

### 2.5. Predictive Marker

In cases when the disease involvement of regional lymph nodes is found in a resected tumor specimen, patients are usually candidates for adjuvant systemic treatment in the form of chemotherapy [1]. Chemotherapy is often also indicated in the case of a metastatic disease [46]. There are many different chemotherapeutic agents that can be combined in many different chemotherapeutic regimes. However, the effect of chemotherapy on a specific patient cannot be predicted. The effect of chemotherapy is assessed only indirectly by repeated imaging following the course of systemic treatment.

The presence of CTCs and ctDNA in the peripheral blood of patients during the course of chemotherapy has been shown to correlate well with the effects of chemotherapy [47]. Specific ctDNA detection has also been used as guidance for specific systemic therapy due to its established concordance with the primary tumor’s RAS mutation status, for it is known, that anti-epidermal growth factor receptor (EGFR) therapy is ineffective in the case of RAS mutations [46,48]. Moreover, the therapy induced genotypic changes observed during systemic treatment, which can render such therapy ineffective, can also be detected from ctDNA [49]. Medical oncology research involving the use of liquid biopsy is for now focusing mainly on assessing the effects of treatment and on the guidance of anti-EGFR therapy and not on the decisions for a specific type of systemic treatment [46,50]. The concept of liquid biopsy, however, harbors great potential as a guiding tool of systemic therapy in the future, although the technology applied would have to be cheap and simple in order to allow its repeated use in hospitals without the state-of-the-art molecular biological laboratories.

### 2.6. Follow-Up

After the completion of treatment, patients are scheduled for regular follow-up visits, for it is known that CRC can recur despite radical surgical resection (and adjuvant chemotherapy) [51]. During the recommended follow-up regimen, certain tumor markers (CEA) and imaging modalities are repeated regularly. These follow-up visits are cost and labor intensive for attending physicians and pose a certain psychological burden for patients because it takes at least five years of uncertainty before they can be declared cured of their disease [52].

Repeated peripheral blood testing has been traditionally used during the course of follow-up visits of patients with CRC. The blood tests applied are based on rather unspecific tumor markers (mainly CEA) with a reasoning that closely resembles the one of liquid biopsy, despite the fact that the clinical benefits of such testing are questionable [53]. Since it is expected that the detection of CTCs/ctDNA is probably more sensitive and specific for the detection of tumor recurrence than CEA alone, liquid biopsy might replace the use of tumor markers during the course of patients’ follow-ups. Such a liquid biopsy test would, however, have to be compared with tumor marker tests also from the point of view of user-friendliness and costs.

## 3. Current Issues of Liquid Biopsy

Despite all the aforementioned potential benefits of liquid biopsy use, clinical cancer medicine has not yet profited so much from the advances of basic science and molecular biology. Crucial clinical decisions in the management of CRC patients are currently still based on the decades-old technology of histopathological analysis of tumor tissue and radiologic imaging with all of their limitations. Although the concept of liquid biopsy seems to have great potential in the management of CRC patients, there are certain practical issues that have to be resolved before it can be used in clinical practice [54].

The first important issue is clearly the fact that only few to no CTCs can be detected within the standard-size blood probes that are currently used in clinical practice. We know from previous research that even patients with an established metastatic disease sometimes have no CTCs in the standard blood probes, let alone the majority of patients with the localized disease [55]. Therefore, the technology based on the detection of CTCs alone is less useful for clinical practice. The only solution to this problem would be the analysis of greater amounts of patients’ blood, but this would probably be too invasive and impractical for clinical use. In addition to that, metastatic tumor cells seem to undergo the process of epithelial–mesenchymal transition, which transforms their phenotype and might render them undetectable by many current CTC detection techniques [56]. Therefore, the detection of ctDNA appears to be a more promising basis for liquid biopsy [9,57,58]. It can be reasonably expected that, due to its smaller size, ctDNA is much more evenly distributed throughout the patient’s bloodstream and can be detected in much smaller blood amounts than CTCs. Yet, it is difficult to determine the standard set of a few typical mutations to ensure the high sensitivity of the test, for it is known that even frequent mutations are not present in all tumors of the same type [59]. Furthermore, each tumor is a genetic heterogeneous population of tumor cells, which complicates the matter even more [60]. The importance of identifying the special set of DNA mutations which is to be used as a liquid biopsy for different tumor types has already been recognized [61]. In a recent study, Cohen et al. have been able to demonstrate the high (69%–98%) sensitivity and >99% specificity of a blood test based on the detection of many different DNA mutations, even in localized, resectable tumors of many different types [62]. New technologies, such as next-generation sequencing (NGS) and digital polymerase chain reaction (dPCR), could further improve ctDNA-based liquid biopsy in the nearest future [63,64,65]. A very interesting concept has already been tested by Reinert et al., who first used NGS in order to detect tumor specific DNA changes in colorectal cancer patients and then dPCR to detect these changes in the peripheral blood. They have found ctDNA-based liquid biopsy superior to CEA assessment in all regards [66].

The aforementioned aspects should be taken into account if the liquid biopsy test is supposed to respect the basic science’s current understanding of tumor biology. To respect the demands of clinical practice, on the other hand, the test has to be user friendly and cheap on top of that.

Currently, the blood probes used for clinical research on liquid biopsy have to be taken to the laboratory and processed quickly or be frozen at low temperatures until processing. This is inconvenient in the clinical setting. The analyses are also labor and cost intensive. Furthermore, there are uncertainties with the used cut-off values, calibration, etc. because there is no standardization of such tests. Therefore, the results of different institutions are not directly comparable to each other, which makes the assessment of the published results impossible.

The solution to this problem could be a simple machine which would automatically and uniformly analyze a patient’s blood without special preparations. The only machine accepted so far by the FDA for clinical use has been the Cellsearch device. It, however, only detects whole tumor cells based on the technology of cell surface markers and is therefore subjected to the aforementioned shortcomings of CTC-based liquid biopsy testing [67]. A much more attractive option is a simple one-way test based on ctDNA (and CTC) detection (similar to well-established pregnancy tests, for example), with positive or negative test results (positive/negative liquid biopsy) only.

Molecular biology and technology science have obviously recognized this need by introducing the concept of lab-on-a-chip (LOC). The concept itself seems very promising for practical use and is currently under investigation in many fields of natural science and also medicine [68,69,70].

## 4. Lab-on-a-Chip and Liquid Biopsy—Future Perspectives

LOC technology seems to be perfectly compatible with the concept of liquid biopsy and could enhance its use in the clinical setting. A droplet of a patient’s blood on a small device which will tell whether or not there are some traits of tumor in the patient’s blood is surely something of which every clinician dealing with oncological patients is dreaming. Yet, once we go a little further into details, things get more complicated. Crucial issues to be resolved in the future are the technical solutions of the chips and their use in the clinical setting.

Technical issues to be resolved apply to what the chips should detect and how. The majority of the publications on LOC so far deal with the detection of whole cells (CTCs). There is a huge variety of technical solutions described in the literature that makes the clinicians almost impossible to choose from [69,71,72,73,74]. LOC technology has also been integrated into an automated device with a higher CTC detection rate in different cancer types, as compared to the Cellsearch device [75]. Yet, as already mentioned, liquid biopsy clinical research has been lately focusing more on ctDNA and not so much on CTCs anymore. However, LOC with DNA-based detection techniques have also been developed [76,77]. Nevertheless, it seems that the technical development of microfluidic chips is not following clinical and basic research demands and needs.

This might also be the reason for only very few reports on the use of LOC in liquid biopsy clinical studies. Only a few publications are available on the subject of LOC and colorectal cancer, although the latter is one of the most common cancer types in the Western world and, as such, the subject of continuous research. In one of the few published clinical studies, Chen et al. have used a specific biomimetic lipid coated microfluidics chip for CTC detection in 54 CRC patients and found a correlation between CTCs and tumor stages, as well as between circulating tumor microemboli and disease progression [78]. In another interesting clinical study, Crotti et al. have used nanoporous silica chips for the detection of specific peptides in the peripheral blood of 33 patients with rectal cancer after neoadjuvant radiochemotherapy and found that they could be used to discriminate between responders and nonresponders [79]. In one of the biggest clinical studies on the subject so far, Tsai et al. detected CTCs in 110 CRC patients of all stages with an anti-EpCAM-SLB coated chip and found a CTC count of >5 per 2 mL of blood to be an independent prognostic factor of distant metastases [80]. Using an interesting miRNA detection technology that uses a droplet microfluidic device, Zhang et al. found the target miRNA content in the blood of three colon cancer patients to be significantly higher than in healthy donors’ blood [81].

A part of the problem for a small number of clinical studies on LOC and liquid biopsy is surely also the publishing policy of scientific journals. Clinical medical journals rarely publish subjects that have no direct or immediate relevance to clinical practice and are interested mainly in practically oriented clinical trials, and not so much in translational research. The scientists engaged in basic/technical research, on the other hand, publish in their own journals and attend their own conferences. In March 2018, the search queries in PubMed on the concept of LOC retrieved around 9400 results, and the queries on liquid biopsy around 3700 results. At the same time, the search of the clinical studies on CRC retrieved more than 211,000 results with only a handful of them on LOC. There is obviously too little interaction between these scientific communities. Sackmann et al. already highlighted this problem in their review on LOC technology in 2014 and predicted that the only way of introducing LOC in clinical studies is by making devices practical and with the close cooperation of engineers and clinicians [82].

## 5. Conclusions

Progress in the field of liquid biopsy can, therefore, only be achieved through the close cooperation of technical scientists, molecular biologists, and clinical physicians. It has been recognized that clinical medicine does not profit from technical advances if the latter are only distributed within the technical community. If technical advances are to become interesting for clinical use, they have to primarily address the needs of clinicians and patients. Only then will clinicians be willing to engage in clinical trials. Through many clinical trials, technical advances might eventually enter into routine clinical practice.

Although not fit for clinical practice yet, the concept of liquid biopsy offers many theoretical advantages over standard CRC assessment methods. With expected technical advances, the current shortcomings of the liquid biopsy concept might be resolved soon. Especially, LOC technology might play an important role in solving the problems regarding practicality, standardization, and the comparisons of results.
